# HOCI Probe CPP Induces the Differentiation of Human Dermal Fibroblasts into Vascular Endothelial Cells through PHD2/HIF-1α/HEY1 Signaling Pathway

**DOI:** 10.3390/cells11193126

**Published:** 2022-10-04

**Authors:** Xiaoling Cui, Jie Wen, Nan Li, Xuxiao Hao, Shangli Zhang, Baoxiang Zhao, Xunwei Wu, Junying Miao

**Affiliations:** 1 Shandong Provincial Key Laboratory of Animal Cells and Developmental Biology, School of Life Science, Shandong University, Qingdao 266237, China; 2 School of Stomatology, Shandong First Medical University & Shandong Academy of Medical Sciences, Jinan 250117, China; 3 Institute of Organic Chemistry, School of Chemistry and Chemical Engineering, Shandong University, Jinan 250100, China; 4 Engineering Laboratory for Biomaterials and Tissue Regeneration, Ningbo Stomatology Hospital, Ningbo 315040, China; 5 Savaid Stomatology School, Hangzhou Medical College, Hangzhou 310058, China; 6 The Key Laboratory of Cardiovascular Remodeling and Function Research, Shandong University Qilu Hospital, Chinese Ministry of Education and Chinese Ministry of Health, Jinan 250012, China

**Keywords:** differentiation, human dermal fibroblasts, vascular endothelial cells, hypochlorous acid probe, PHD2/HIF-1α/HEY1 signal pathway

## Abstract

Human dermal fibroblasts (HDFs) have the potential to differentiate into endothelial cells (VECs). In our previous research, we reported that a hypochlorous acid (HOCl) probe CPP efficiently induced the differentiation of HDFs into VECs, however, the mechanism of differentiation was not clear. As an HOCI probe, CPP binds HOCI to modulate its effects. In this study, through Western blotting, qPCR, and PHD2 enzyme activity assay, we found that CPP inhibited the enzyme activity of prolyl-4-hydroxylase 2 (PHD2), thereby stabilizing HIF-1α. To further clarify the mechanism by which CPP inhibits PHD2 enzyme activity, we constructed plasmids, and found that CPP inhibited PHD2 activity to increase the HIF-1α level through the modulation of PHD2 at Cys302 by HOCl in HDFs. Furthermore, RNA-seq experiments showed that CPP could induce the expression of HEY1, which is not only a target gene regulated by HIF1α, but also a key transcription factor for VECs. We used siRNA transfection and in vivo experiments to confirm that CPP could induce HDFs to differentiate into VECs by HEY1. In summary, we identified a new inhibitor of PHD2, demonstrated the new role of HOCl in cell differentiation, and elucidated the mechanism by which HOCl probe CPP induced the differentiation of HDFs into VECs.

## 1. Introduction

Over the years, researchers have demonstrated that human dermal fibroblasts (HDFs) can differentiate into vascular endothelial cells (VECs) through reprogramming technology [1]. Previously, we found that the small chemical molecule CPP ((E)-4-(4-(4-(7-(diethylamino)-2-oxo-2H-chromene-3-carbonyl) piperazin-1-yl) styryl)-1-methylpyridin-1-ium iodide) could induce HDFs to differentiate into vascular endothelial cells [2] (In press; Identification of a new way to induce differentiation of dermal fibroblasts into vascular endothelial cells; Stem Cell Research & Therapy). However, there was no clear mechanism by which HDFs differentiate into VECs. As a hypochlorous acid (HOCl) probe, CPP is suitable for studying HDF differentiation into VECs.

HOCl is produced by the catalytic action of myeloperoxidase (MPO), a heme peroxidase released by activated neutrophils that uses chloride anions and H2O2 as substrates [3]. The role of HOCl is a major component of reactive oxygen species (ROS). Current research shows that HOCl can modulate signal transduction and cell fate by modifying proteins [4,5,6]. However, it is unclear whether HOCl plays a vital role in regulating cell differentiation. In previous studies in our lab, we found that an endoplasmic reticulum-targeted hypochlorous acid probe (ZBM-H) inhibited the oxidation of Grp78 at lysine 353 by binding HOCl [7]. In this study, as an HOCl probe and an inducer of HDFs differentiation into VECs, CPP will be used to bind HOCl to study its impact on differentiation.

Studies have shown that hypoxia can promote the reprogramming of dermal fibroblasts into iPSCs [8], and the combined treatment of hypoxia and ETV2 can induce differentiation of dermal fibroblasts into endothelial progenitor cells [9]. Hypoxia-inducible factor-1α (HIF-1α) is a nuclear protein with transcriptional activity, that can regulate the expression of a series of target genes, including vascular endothelial growth factor (VEGF), angiogenin (ANGPT), fibroblast growth factor 2 (FGF-2), placental growth factor (PlGF), platelet-derived growth factor BB (PDGF-BB), HEY1, etc., and then regulate the survival, migration and proliferation of vascular endothelial cells [10,11]. Prolyl hydroxylase domain proteins (PHDs) are capable of hydroxylating HIF-1α on its oxygen-dependent degradation domain (ODDD), which can promote the degradation of HIF-1α, and result in reduced VEGF transcription and inhibition of angiogenesis [12]. Some studies have shown that the formation of inter or intramolecular disulfide bonds in the cysteine326 of PHD2 may affect its enzymatic function. Except for Cys326, Cys302 and Cys323 of PHD2 may also undergo oxidative modification, but further proof is needed [13,14].

Similar to the HIF signaling pathway, the Notch signaling pathway is a crucial regulator of gene expression, including genes that regulate cell differentiation. Studies have shown that HIF-1α is an inducer of Notch signaling. HEY1, a member of the Notch signaling pathway and a target gene of HIF-1α, plays an essential role in maintaining endothelial cell function [15]. Studies have shown that the Hey1 deficiency causes lethal anomalies of the thoracic great vessels [16]. However, it is unclear whether HEY1 is crucial for the transformation of HDFs into VECs. In this study, we found that the HOCl probe CPP could bind HOCl, inhibit the oxidation of PHD2 at cysteine 302, and raise the protein level of HIF1a, which promoted the expression of HEY1 and caused the differentiation of dermal fibroblasts into vascular endothelial cells. These results provide new ideas for the study of dermal fibroblast differentiation.

## 2. Materials and Methods

### 2.1. Antibodies

Antibodies against VEGF (sc-7269), FGF-2 (sc-271847) were obtained from Santa Cruz Biotechnology (Santa Cruz, CA, USA). Antibodies against HIF-1α (20960-1-AP) and Collagen 1 (14695-1-AP) were obtained from Proteintech group (Wuhan, China). Antibodies against CD31 (A19014) and Vimentin (A19607) were obtained from ABclonal (Wuhan, China). Antibodies against CD133 (64326S) were obtained from Cell Signaling Technology (Danvers, MA, USA). Antibodies against HEY1 (ab154077) were obtained from Abcam (Cambridge, U.K.). The antibodies against β-actin were obtained from Sigma-Aldrich (St. Louis, MO, USA). Horseradish peroxidase-conjugated secondary antibodies were obtained from Jackson Immunoresearch (West Grove, PA, USA). The secondary antibodies used for immunofluorescence were donkey anti-rabbit which was obtained from IgG Alexa Fluor-546 (A-11037; Invitrogen, Carlsbad, CA, USA).

### 2.2. Cell Culture

Human primary HDFs were derived from the foreskin of children, and were isolated according to our previous publication [17]. Briefly, the skin was cut into 5 × 5 mm strips and incubated overnight at 4 °C in phosphate-buffered saline (PBS) (2.5 mg/mL; Sigma Chem. Co). On the following day, forceps were used to separate the epidermis and dermis. After cutting the dermis with crossed scalpels, we incubated it in collagenase solution (2.5 mg/mL, 37 °C) for 30 min. Next, the dermal solution was neutralized in Dulbecco’s modified Eagle’s medium (DMEM) with 10% fetal bovine serum (FBS), passed through 100 μm filter, and the filtrate centrifuged and rinsed with PBS. A pellet of dermal cells was plated in DMEM/F12 (3:1) supplemented with 2% B27 supplement, 5% FBS, 40 mg/mL fungizone, 40 ng/mL FGF2, 40 ng/mL EGF and 40 ng/mL FGF2; at 80–90% confluence, dermal cells were passaged. HDFs were maintained in DMEM Basic medium (C11995500BT, Gibco, Grand Island, NY, USA) supplemented with 10% (*v*/*v*) bovine calf serum. All cell lines were cultured at 37 °C in a 5% CO_2_ atmosphere. All cell lines were checked by DNA short tandem repeat (STR) profiling and were confirmed to be mycoplasma negative. In this study, the population doubling level (PDL) of cultured HDFs was less than 10 (PDL < 10).

### 2.3. Western Blot Analysis

Cells were treated differently before adding lysate (Beyotime, Shanghai, China). Cell lysates were centrifuged at 12,000 rpm for 15 min. The concentration of the supernatant was tested using a BCA protein assay kit (Beyotime, Shanghai, China). Next, cell lysates (30 μg protein per lane) with a loading buffer were separated by SDS-PAGE, and the proteins were transferred to polyvinylidene difluoride membranes. After that, the membranes were incubated with the primary antibodies at 4 °C overnight. The next day, membranes were incubated with the secondary antibodies at room temperature for 1 h. An enhanced chemiluminescence detection kit (34080, Thermo Fisher, Waltham, MA, USA) was used to detect antibodies bound to proteins. Relative quantities of specific bands were analyzed by Image J software and were normalized to loading controls.

### 2.4. Quantitative Real-Time PCR

RNA was isolated using the Trizol reagent method (Takara, Tokyo, Japan), and extracted total RNAs were obtained by reverse cDNA by the PrimeScript RT reagent kit with gDNA Eraser (Takara). PCR reactions involved the use of SYBR Premix Ex Taq (Tli RNaseH Plus, Takara) and levels of expressed genes were measured by the 2^−ΔΔCt^ method with MxPro 4.00 (Stratagene, La Jolla, CA, USA). Table S1 lists the primers used.

### 2.5. Co-Localization of CPP and Prolyl-4-hydroxylase 2 (PHD2) or HIF-1α

Cells were treated with CPP (10 μM) for 3, 6, 12, 24 and 48 h. The treated cells were fixed in 4% paraformaldehyde for 30 min at room temperature and then washed twice with PBS for 5 min each. Cells were permeabilized with 0.2% Triton-X100 for 2 min, then blocked with 10% normal donkey serum for 30 min, and then incubated with the primary antibodies at 4 °C overnight. Cells were then incubated with the secondary antibodies (1:200) for 1 h at 37 °C, and fluorescence was detected using a laser scanning confocal microscope Zeiss LSM700 (Jena, Germany).

### 2.6. PHD2 Enzyme Activity Assay

The treated HDFs were digested with 0.25% trypsin and then were disrupted by repeated freeze–thaw cycles and centrifuged at 3000 rpm for 20 min. The supernatants were collected, and PHD2 enzyme activity was tested using a human prolyl hydroxylase 2 (PHD2) ELISA detection kit (JL47645, J&L Biological Industrial Co., Ltd., Shanghai, China).

### 2.7. Plasmids and Overexpression

Full-length PHD2 was cloned into the pCDNA3.1 Zeo (+) plasmid (pCDNA-PHD2). His6-tagged PHD2 plasmids, including his6-PHD2-wt (wild-type), his6-PHD2-mut1 (C302A) (mutant), his6-PHD2-mut2 (C323A) (mutant) and his6-PHD2-mut3 (C326A) (mutant), were constructed. HDFs at 70–80% confluence were transfected with those expression vectors for 24 h using Lipofectamine 2000 (11668–019, Invitrogen, Waltham, MA, USA) according to the manufacturer’s instructions. The cells were then treated with CPP for 48 h and then harvested and analyzed by Western blot assay.

### 2.8. siRNAs Transfection

Duplex oligonucleotides were chemically synthesized and purified by Tsingke Biotechnology (Beijing, China). The small interfering RNA (siRNA) duplexes used were HEY1:siHEY1-1: 5′-CAGAAGUUGCGCGUUAUCUTT-3′ (forward) and 5′-AGAUAACGCGCAACUUCUGTT-3′(reverse); siHEY1-2: 5′-GACUGGUUUCGCAUCUCAATT-3′ (forward) and 5′-UUGAGAUGCGAAACCAGUCTT-3′(reverse). Cells were transfected with siRNA duplexes using Lipofectamine 2000 (11668-019, Invitrogen, Waltham, MA, USA) according to the manufacturer’s instructions. The cells were changed to a fresh complete medium after 4–6 h, followed by CPP treatment. The above operations were repeated every 48 h, and protein and RNA were extracted for relevant detection after being repeated three times.

### 2.9. RNA Sequencing Analysis

Total RNA from DMSO-treated HDFs and CPP-treated HDFs (6 days) was subjected to RNA sequencing analysis (RNA-seq). RNA-seq was conducted by Novogene. An index of the reference genome was built using Hisat2 v2.0.5 and paired-end clean reads were aligned to the reference genome using Hisat2 v2.0.5. Differential expression analysis was performed using the DESeq2 R package (1.20.0). The *p* values were adjusted using the Benjamini and Hochberg method. Corrected *p*-value of 0.05 and absolute fold change of 2 were set as the threshold for significantly differential expression.

### 2.10. Colocalization between Different Proteins In Vivo

A total of 15, five-week-old and pathogen-free C57BL mice with a mean weight of 20 g were randomly divided into three groups: (1) a PBS injection group; (2) a CPP (1 mg/kg/day) injection group; and (3) a CPP (10 mg/kg/day) injection group. After two weeks of continuous intraperitoneal injections, the mice were sacrificed and their skin tissues were embedded in paraffin. Immunofluorescence analysis was conducted to analyze the co-localization between HEY1 (ABclonal, Wuhan, China) and Vimentin (ABclonal, Wuhan, China), CDH5 (ABclonal, Wuhan, China) and Vimentin, or CD31 (ABclonal, Wuhan, China) and Vimentin, in mouse skin. DAPI was indicated to the nucleus. Next, ImageJ was used to analyze the co-localization of the different proteins.

### 2.11. Statistical Analysis

Data were reported as means ± SE from at least three separate experiments and were analyzed by *t*-test with SPSS 17.0 (SPSS Inc., Chicago, IL, USA). Student’s *t*-test was performed to compare the mean between two groups. One-way ANOVA followed by multiple comparisons was used for comparison between more than two groups. Differences with a *p* < 0.05 were recognized as statistically significant.

## 3. Results

### 3.1. CPP Induces the Differentiation of HDFs into Vascular Endothelial-Like Cells through Increases of HIF-1α

**Figure 1:**
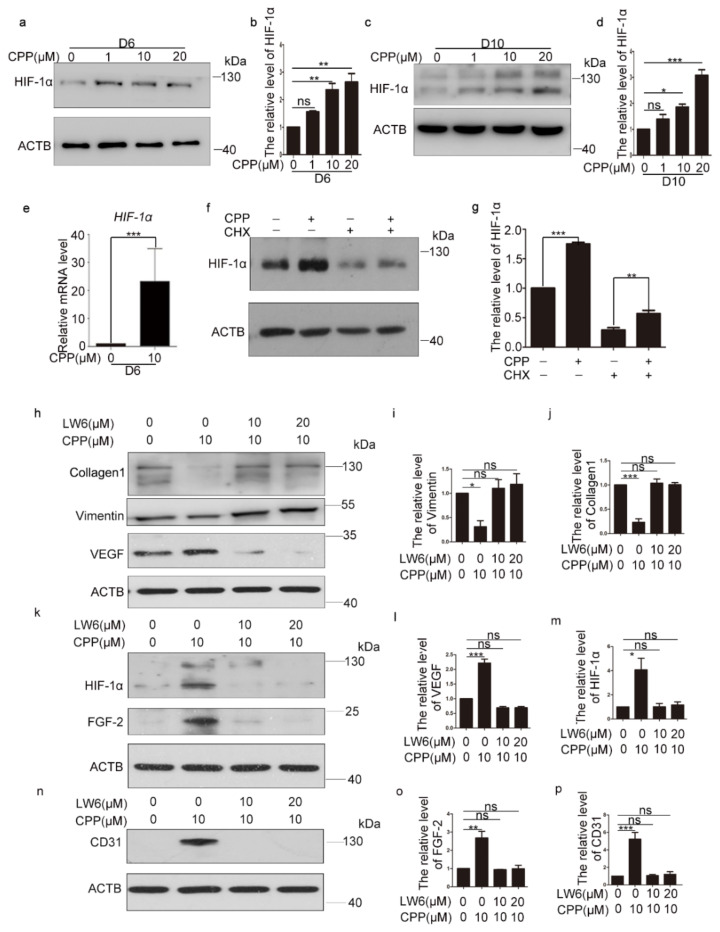
**CPP induces the differentiation of HDFs into vascular endothelial cells by increasing levels of HIF-1α.** (**a**–**d**) HDFs were treated with CPP (0, 1, 10 or 20 μM) for 6 days (D6) or 10 days (D10), after which Western blot analysis was used to measure the protein level of HIF-1α. β-actin (ACTB) was used as a loading control. (**e**) HDFs were treated with CPP (0 or 10 μM) for 6 days (D6), after which qRT-PCR was used to detect the mRNA level of HIF-1α. (**f,g**) In the presence or absence of cycloheximide (CHX, 10 μg/mL), HDFs were treated with 10 μM CPP for 48 h, after which the protein level of HIF-1α was detected by Western blot. β-actin (ACTB) was used as a loading control. (**h**–**p**) HDFs were pretreated with LW6, an inhibitor of HIF-1α, for 12 h, and then treated with CPP (10 μM) for 6 days (D6), and protein levels of CD31, HIF-1α, VEGF, FGF-2, Collagen 1 and Vimentin were determined by Western blot. β-actin (ACTB) was used as a loading control. Data are presented as means ± SEM, * *p* < 0.05, ** *p* < 0.01, *** *p* < 0.001, *n* = 3.

HIF-1α regulates the expression of nearly 200 genes involved in biological processes, and, therefore, is a key regulator in vascular endothelial cells [18]. Hypoxia is known to induce HIF-1α levels, and more and more studies have confirmed that HIF-1α could regulate stem cell differentiation into endothelial cells [19]. To elucidate the mechanism of HDFs differentiation into vascular endothelial cells, we examined changes in HIF-1α levels during CPP-induced differentiation of HDFs into vascular endothelial cells. First, we treated HDFs with CPP at 1, 10 and 20 μM for 6 days or 10 days, and found that CPP significantly increased the protein level of HIF-1α by Western blot analysis (Figure 1a–d).

Through the above experiments, we found that CPP up-regulated the protein level of HIF-1α, but the mechanism was not clear. In order to understand how CPP increased the protein level of HIF-1α, we tested and found an increased mRNA level of HIF-1α in CPP-treated cells (Figure 1e). Interestingly, we also observed that CPP increased the protein level of HIF-1α even in the presence of the protein synthesis inhibitor cycloheximide (CHX) (Figure 1f,g), which showed that CPP promoted the increase in the HIF-1α protein level, at least in part by inhibiting its degradation.

**Figure 2:**
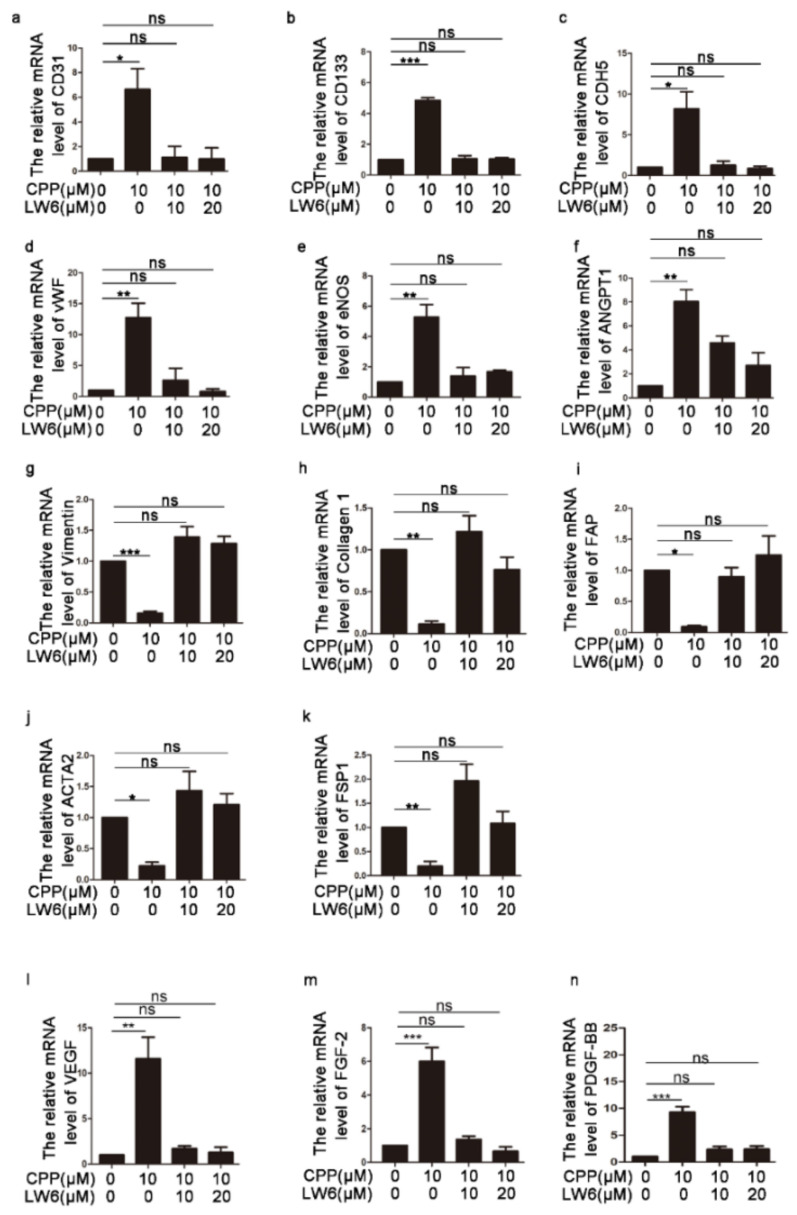
**The effect of CPP on related genes after treatment with an inhibitor of HIF-1α.** (**a**–**n**) HDFs were pretreated with LW6, an inhibitor of HIF-1α, for 12 h, and then treated with CPP (10 μM) for 6 days (D6), and qPCR was conducted to detect the expression of EC genes (CD31, CD133, CDH5, vWF, eNOS and ANGPT1), fibroblastic genes (Vimentin, Collagen 1, FAP, ACTA2 and FSP1), and pro-angiogenic factors (FGF-2, VEGF, PDGF-BB). Data are presented as means ± SEM, * *p* < 0.05, ** *p* < 0.01, *** *p* < 0.001, *n* = 3.

Next, we investigated whether HIF-1α is involved in the differentiation of HDFs into endothelial-like cells induced by CPP, and treated dermal fibroblasts with CPP combined with the HIF-1α inhibitor LW6. We treated HDFS with CPP with or without LW6 to conduct Western blot. Compared with DMSO-treated HDFs, the protein level of EC marker protein CD31, and pro-angiogenic factors HIF-1α, FGF-2, VEGF, fibroblastic marker proteins Vimentin and Collagen 1, were markedly changed in the group of CPP-treated HDFS, but LW6 significantly abolished the changed protein levels induced by CPP (Figure 1h–p). Furthermore, we tested the expression levels of EC genes (CD31, CD133, CDH5, vWF, eNOS and ANGPT1), fibroblastic genes (Vimentin, Collagen 1, FAP, ACTA2 and FSP1), and pro-angiogenic factors (FGF-2, VEGF, PDGF-BB), and obtained the same results (Figure 2). In summary, these data suggested that CPP induced the differentiation of HDFs into endothelial-like cells by promoting an increased protein level of HIF-1α.

### 3.2. CPP Inhibits the Activity of PHD2, which Positively Regulates the Degradation of HIF-1α, by Binding to Free HOCl

**Figure 3:**
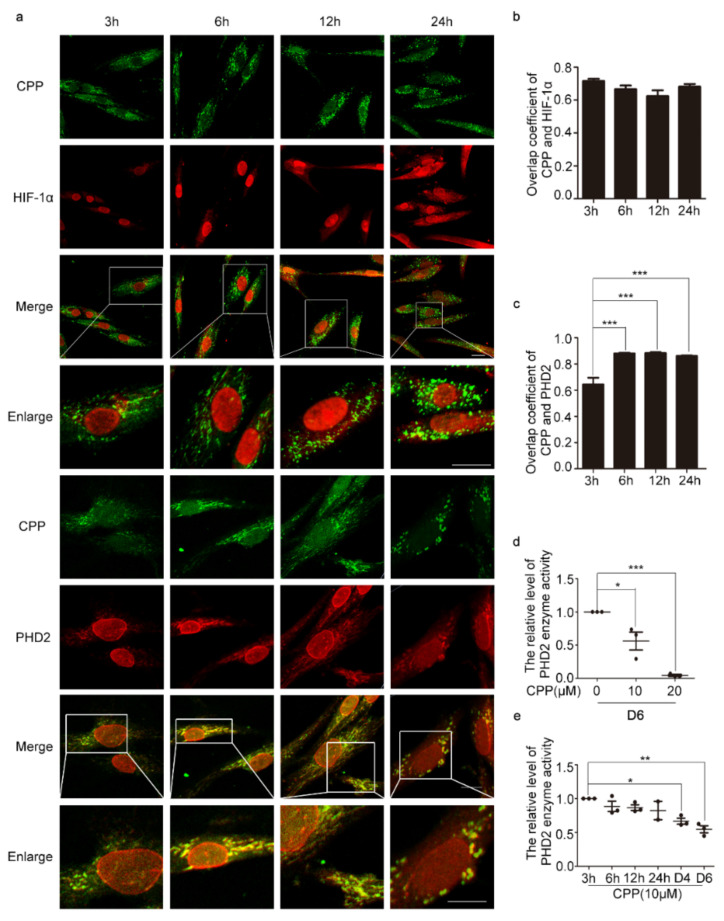
**CPP inhibits PHD2 activity in HDFs.** (**a**–**c**) HDFs were treated with CPP (10 μM) for 3, 6, 12 and 24 h, after which immunofluorescence was used to detect the co-localization of CPP and HIF-1α, or CPP and PHD2. Scale bars: 20 μm. (**d**,**e**) CPP inhibited PHD2 activity in a dose- and time-dependent manner. HDFs were treated with CPP at 0, 10 or 20 μM, for 6, 12, 24 h, or 4 days (D4) or 6 days (D6), respectively, after which the effects of CPP on PHD2 activity were measured using a PHD2 enzyme-linked immunoassay kit. Data are presented as means ± SEM, * *p* < 0.05, ** *p* < 0.01, *** *p* < 0.001, *n* = 3.

As a probe targeting HOCl, CPP can auto-fluoresce in cells [2]. In order to further study whether CPP directly interacts with HIF-1α to up-regulate the protein level of HIF-1α, immunofluorescence was used, and showed that the overlap coefficient of CPP and HIF1a was around 0.6, which is an extremely low value. Therefore, we deduced that CPP did not directly regulate the function of HIF-1α (Figure 3a,b).

Previous studies have revealed that prolyl-4-hydroxylase 2 (PHD2) can utilize oxygen to hydroxylate and target the alpha subunit of HIFs for proteasomal degradation [20,21]. Interestingly, we observed that CPP co-localized with PHD2 in HDFs after 6, 12 and 24 h of treatment with CPP, while the overlap coefficient was only 0.64 at 3 h of treatment with CPP (Figure 3a,c). Next, we found that CPP significantly inhibited PHD2 activity in a dose- and time-dependent manner (Figure 3d,e). Taken together, these data indicated that CPP not only co-localizes with PHD2, but also inhibits its activity.

**Figure 4:**
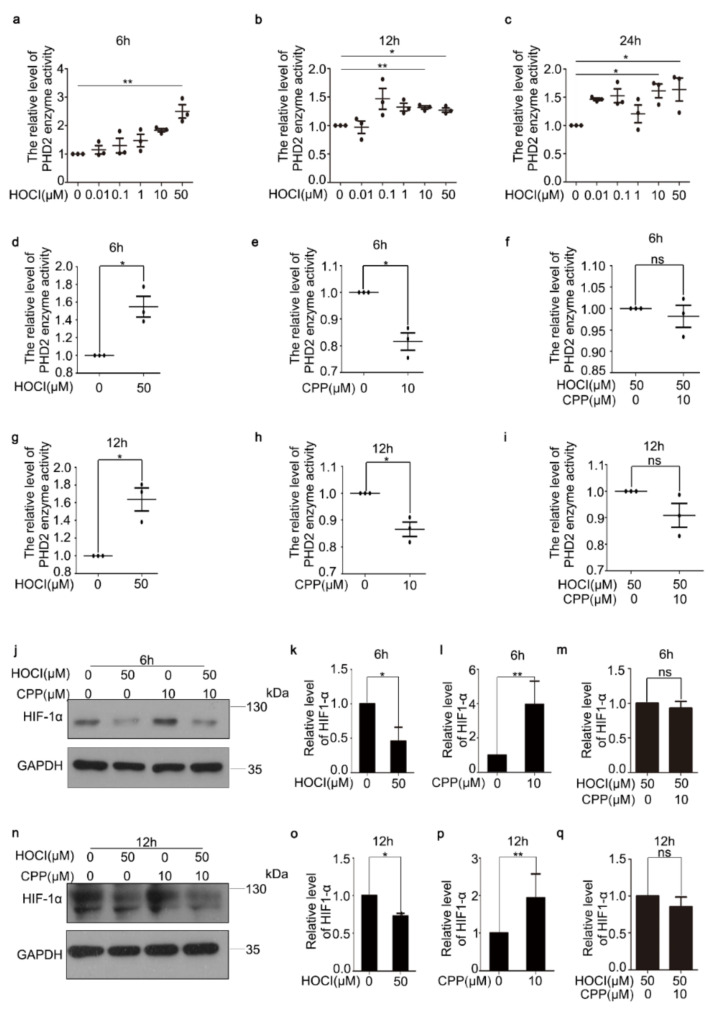
**Exogenous hypochlorous acid partially reversed the CPP-inhibited activity of PHD2 and the CPP-increased protein level of HIF1a.** (**a**–**c**) HOCl activates PHD2. PHD2 activity was examined using a PHD2 enzyme-linked immunoassay kit in HDFs treated with HOCl (0, 0.01, 0.1, 1, 10 or 50 μM) for 6, 12 or 24 h, respectively. (**d**–**i**) HDFs were pretreated with HOCl (50 μM) for 6 or 12 h, and then were treated with CPP (10 μM) for 48 h, after which PHD2 activity was examined using a PHD2 enzyme-linked immunoassay kit. (**j**–**q**) Protein levels of HIF-1α were examined by Western blot. Data are presented as means ± SEM, * *p* < 0.05, ** *p* < 0.01, *n* = 3.

Previously, the HOCl probe ZBM-H demonstrated that it could bind HOCl to inhibit lysine 353 oxidation of Grp78 [7], and the H2S probe CPC demonstrated that it could scavenge endogenous H2S to regulate protein modification [22]. In order to understand how CPP inhibits the activity of PHD2, we hypothesized that CPP could bind to HOCl and inhibit the function of PHD2. To test that hypothesis, cells were incubated with HOCl at different concentrations for 6, 12 and 24 h and then were analyzed for PHD2 activity. The data showed that the addition of HOCl significantly activated PHD2 at 6, 12 and 24 h (Figure 4a–c). HDFs were treated with or without HOCl (50 μM) for 6 and 12 h, and were then treated with or without CPP (10 μM) for 48 h, after which the enzyme activity of PHD2 was detected. The results showed that CPP did not inhibit the enzyme activity of PHD2 in the presence of exogenous HOCl (Figure 4d–i). Similarly, we tested the protein level of HIF-1α and obtained the same results (Figure 4j–q).

### 3.3. CPP Inhibits PHD2 Enzyme Activity by Inhibiting the Oxidation of PHD2 at Cys302

**Figure 5:**
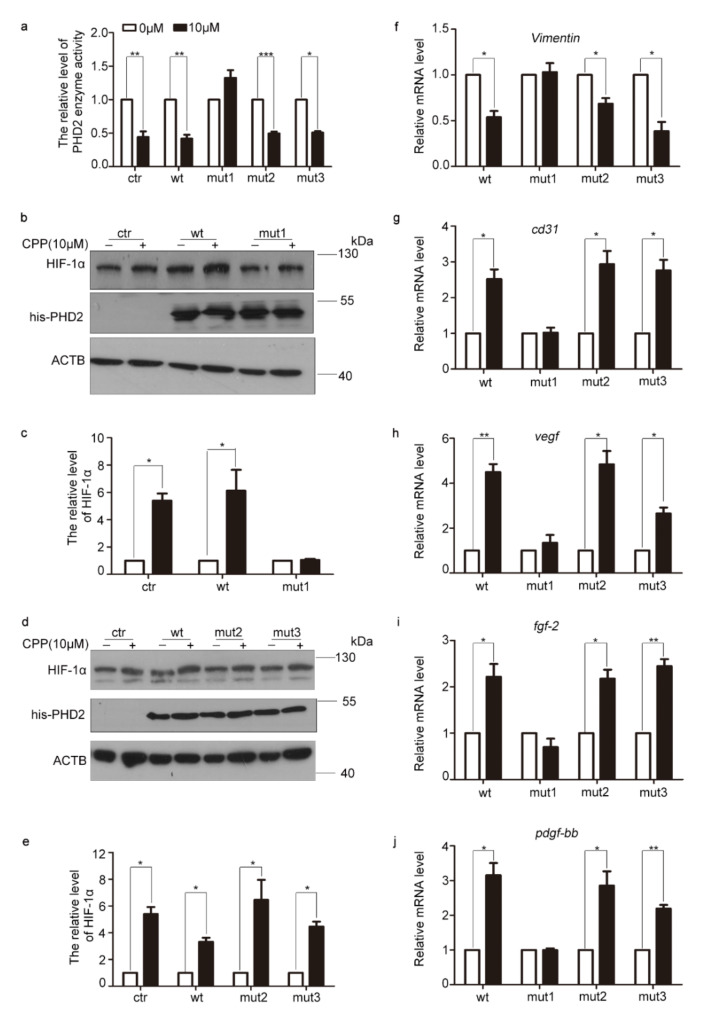
**CPP induces HDFs to differentiate into vascular endothelial cells by inhibiting the oxidation of PHD2 at Cys302.** (**a**) HDFs were transfected with pCDNA-his6 (ctr), pCDNA-his6-PHD2 (wt), pCDNA-his6-PHD2-C302A (mut1), pCDNA-his6-PHD2-C323A (mut2) and pCDNA-his6-PHD2-C326A (mut3) for 24 h, and then were treated with CPP (10 μM) for 48 h, after which the activity of PHD2 was tested using a human PHD2 ELISA detection kit. (**b**–**e**) Western blot analysis of the protein level of HIF-1α in HDFs transfected with pCDNA-his6-PHD2 (wt), pCDNA-his6-PHD2-C302A (mut1), pCDNA-his6-PHD2-C323A (mut2) or pCDNA-his6-PHD2-C326A (mut3) plasmids and treated with CPP (10 μM). β-actin (ACTB) was used as a loading control. (**f**–**j**) HDFs were transfected with pCDNA-his6 (ctr), pCDNA-his6-PHD2 (wt), pCDNA-his6-PHD2-C302A (mut1), pCDNA-his6-PHD2-C323A (mut2) or pCDNA-his6-PHD2-C326A (mut3) for 24 h, and were then treated with CPP (10 μM) for 48 h, after which the mRNA levels of Vimentin, CD31, VEGF, FGF-2 and PDGF-BB were analyzed by qRT-PCR. Data are presented as means ± SEM, * *p* < 0.05, ** *p* < 0.01, *** *p* < 0.001, *n* = 3.

Previous studies have reported that cysteines 302, 323 and 326 of PHD2 can be oxidized [13]. To illustrate how the oxidation modification of those three cysteines of PHD2 might affect its activity, we transfected his6-PHD2-wt (wild-type), his6-PHD2-mut1 (C302A) (mutant), his6-PHD2-mut2 (C323A) (mutant) and his6-PHD2-mut3 (C326A) (mutant) plasmids into dermal fibroblasts and then treated those cells with or without 10 μM CPP. The results revealed that CPP significantly inhibited PHD2 enzyme activity in the presence of his6-PHD2-wt as well as his6-PHD2-mut2 and his6-PHD2-mut3, but not in the presence of his6-PHD2-mut1 (Figure 5a). Further, we investigated the protein level of HIF-1α, the downstream protein of PHD2, and found that CPP was not capable of enhancing the protein level of HIF-1α in the presence of his6-PHD2-mut1. However, CPP did increase the protein level of HIF-1α in the presence of his6-PHD2-wt as well as his6-PHD2-mut2 and his6-PHD2-mut3 (Figure 5b–e).

Next, in order to explore in depth whether Cys302 of PHD2 affects the CPP-induced differentiation of HDFs into vascular endothelial-like cells, we transfected those plasmids into HDFs and then treated those cells with or without 10 μM CPP for 48 h. qRT-PCR analysis revealed that the mutation at cysteine 302 of PHD2 not only inhibited the decrease in Vimentin mRNA level induced by CPP, but also inhibited the increase in CD31, VEGF, FGF-2 and PDGF-BB mRNA levels (Figure 5f–j). Taken together, these data suggested that CPP reduces PHD2 activity to increase the HIF-1α level through the modulation of PHD2 at cysteine 302 by HOCl in HDFs, which results in the differentiation of HDFs into vascular endothelial cells.

### 3.4. RNA-seq Showed That CPP Induces the Expression of Endothelial Cell Transcription Factor HEY1

**Figure 6:**
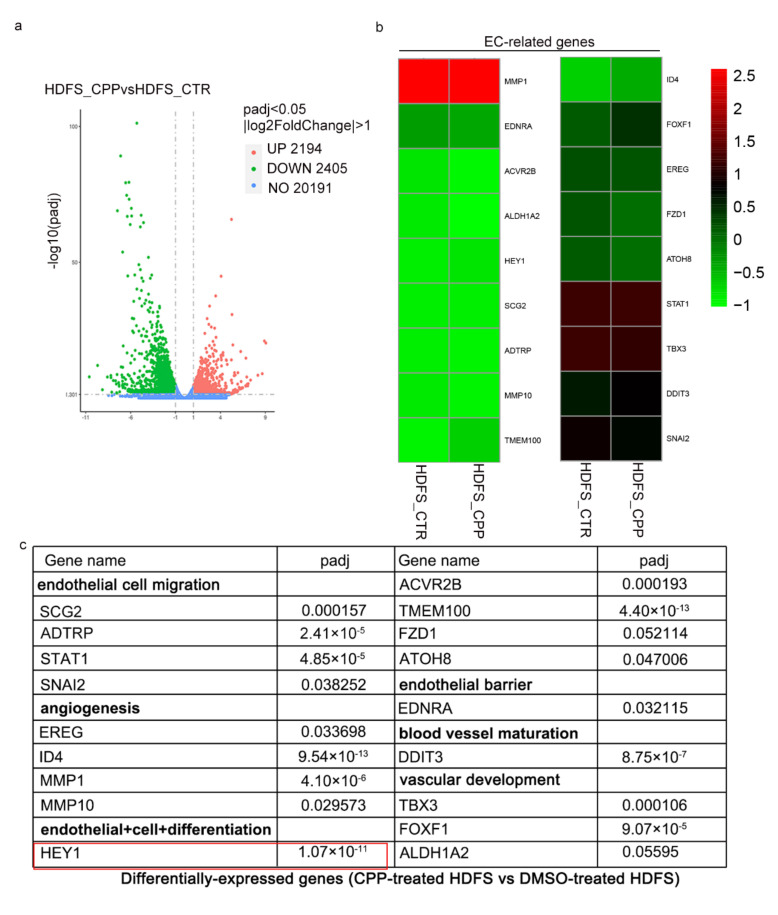
**Transcriptome analysis on CPP-treated HDFs and DMSO-treated HDFs via RNA sequencing.** HDFs were CPP treated for 6 days, RNA-seq was conducted to detect the gene expression. (**a**) Differential gene volcano map. The abscissa is the log2FoldChange value, the ordinate is -log10padj or -log10pvalue, and the blue dotted line represents the threshold line of the differential gene screening criteria. (**b**) Heatmap representing EC-related genes. Expression values relative to the average expression values across all samples are represented by colors from green to red (log2 scale). (**c**) Differential genes related to endothelial cells in CPP-induced HDFs obtained by RNA-seq.

To reveal the molecular mechanism associated with CPP-induced endothelial differentiation, a DEG analysis was performed to identify gene expression changes between HDFs and induced VECs. A total of DEGs (|fold change|>2) were detected between the DMSO-treated HDFs and CPP-treated HDFs cDNA libraries, of which 1171 genes were upregulated, and 1763 genes were downregulated (Figure 6a). This result indicated the great alterations in gene expression during CPP-treated HDFs. By reviewing the literature, we found 18 endothelial cell-related genes with significant differences, which play a role in regulating endothelial cell function (Figure 6b–c). Indeed, we selected 18 significantly elevated VECs-related genes from the CPP-treated HDFs and found that these genes were involved in endothelial cell migration, endothelial barrier, angiogenesis, vascular development and maturation. Among them, endothelial cell transcription factor HEY1 showed a significant difference in expression.

### 3.5. CPP Induces the Differentiation of HDFs into VECs by Promoting the Expression of HEY1, a Key Transcription Factor of VECs

**Figure 7:**
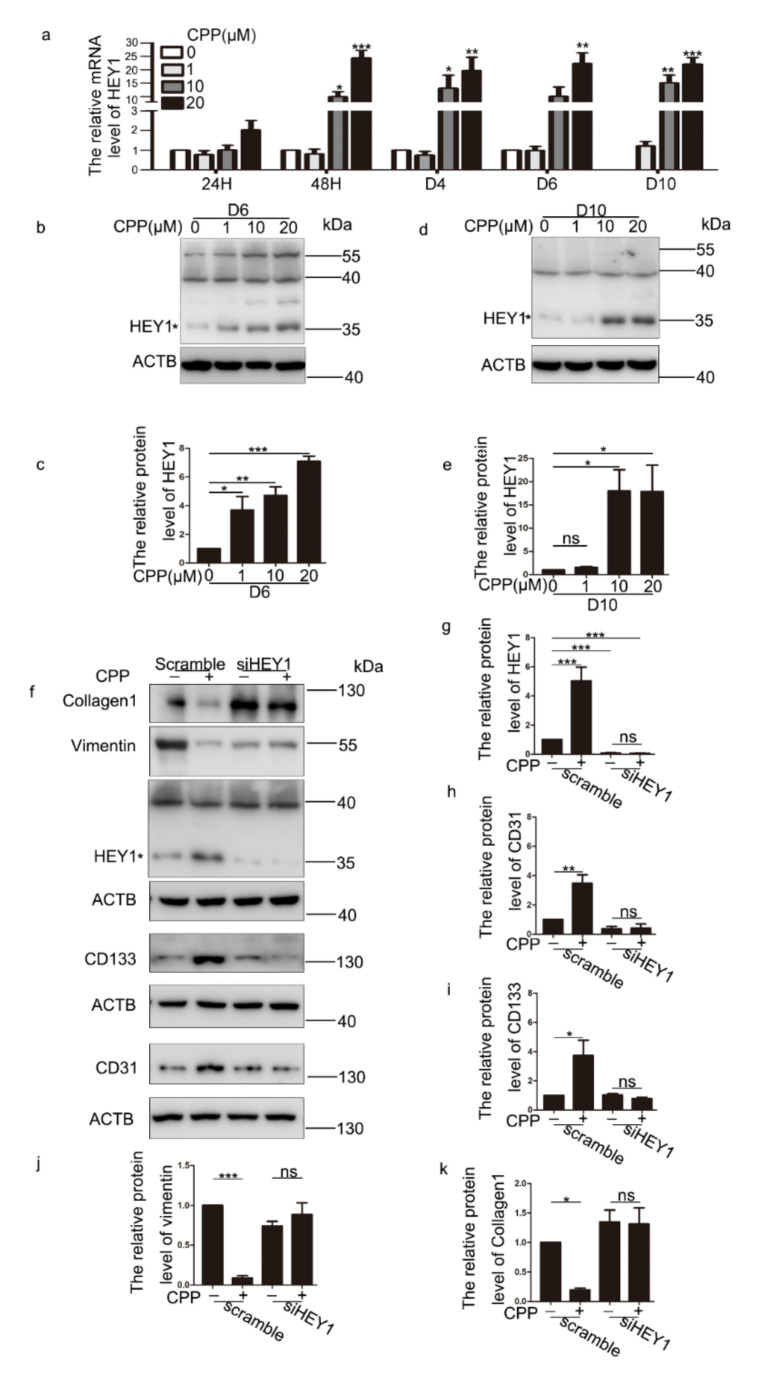
**CPP induces the differentiation of HDFs into vascular endothelial-like cells by promoting the expression of HEY1.** (**a**–**e**) HDFs were treated with CPP (0, 1, 10 or 20 μM) for 24 h, 48 h, 4 days (D4), 6 days (D6) and 10 days (D10), respectively, then qPCR and Western blot were conducted to verify the expression of HEY1. (**f**–**k**) After knocking down HEY1 (siHEY1-1: 40 nM), HDFs were treated with CPP (10 μM) for 6 days, and Western blot was used to detect the protein levels of HEY1 (33 kDa), CD31, CD133, Collagen 1 and Vimentin. Higher-molecular weight bands may represent post-translationally modified HEY1 proteins. β-actin (ACTB) was used as a loading control. Data are presented as means ± SEM, * *p* < 0.05, ** *p* < 0.01, *** *p* < 0.001, *n* = 3.

Studies have shown that HEY1 is a key transcription factor for the maintenance of VECs. Therefore, we used different concentrations of CPP to treat HDFs for 24 h, 48 h, D4, D6 and D10, and then qPCR to verify the expression of HEY1. We found that starting from 48 h, the expression level of HEY1 increased (Figure 7a). Similarly, we tested the protein level of HEY1 at D6 and D10 and obtained the same results (Figure 7b–e).

**Figure 8:**
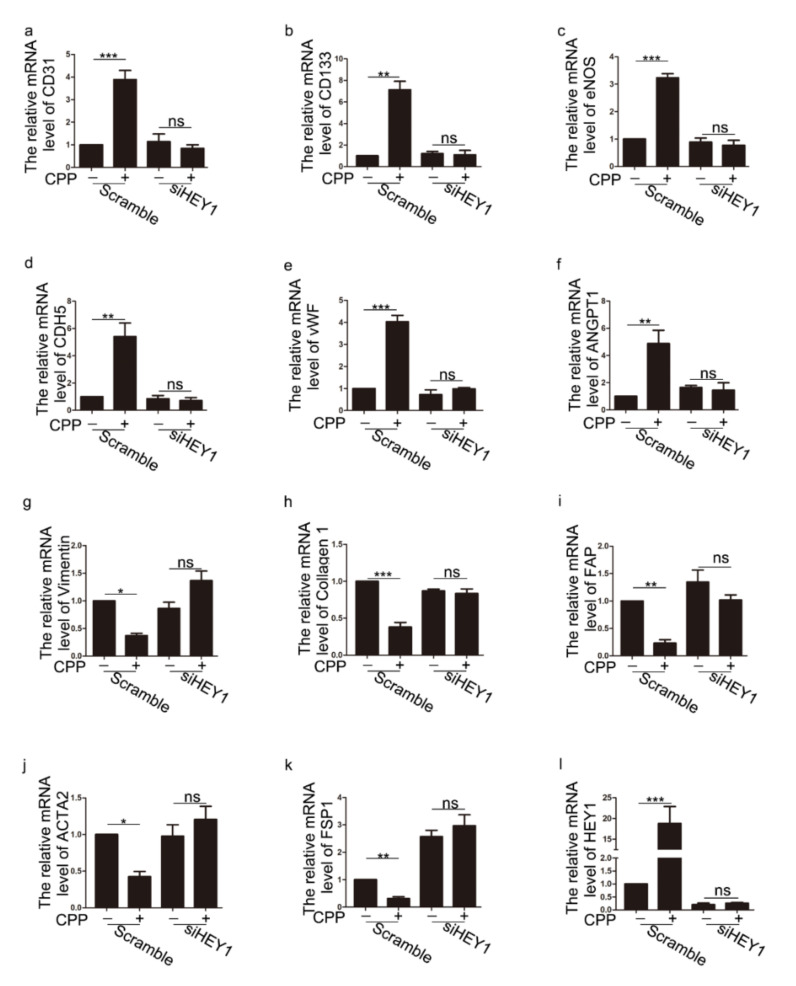
**The effect of CPP on related genes after knocking down HEY1.** After knocking down HEY1 (siHEY1-1:40 nM), HDFs were treated with CPP (10 μM) for 6 days, and then the levels of vascular endothelial cell-related genes (CD31, CD133, CDH5, vWF, eNOS and ANGPT1) (**a**–**f**), and HDFs-related genes (Vimentin, Collagen 1, FAP, ATA2 and FSP1) (**g**–**k**), and HEY1 (**l**), were analyzed by qPCR. Data are presented as means ± SEM, * *p* < 0.05, ** *p* < 0.01, *** *p* < 0.001, *n* = 3.

Next, we investigated whether HEY1 is involved in the differentiation of dermal fibroblasts into endothelial-like cells induced by CPP. We knocked down the mRNA of HEY1 (Supplementary Figure S1). We detected the protein level of the markers of HDFs and VECs. Western blot analysis revealed that siHEY1 significantly abolished the changed protein levels of endothelial cell-associated proteins (CD31and CD133) and fibroblastic marker proteins (Vimentin and Collagen 1) induced by CPP (Figure 7f–k). We also performed qPCR to analyze the expression of VECs-related genes (CD31, CD133, eNOS, CDH5, vWF and ANGPT1) and HDFs-related genes (Vimentin, Collagen 1, FAP, ACTA2 and FSP1). Consistently, when we knocked down HEY1, CPP failed to regulate those genes (Figure 8). Taken together, these data suggest that HEY1 plays an important role in the differentiation of HDFs into VECs.

### 3.6. CPP Promoted Increased Protein Levels of HEY1 in HDFs In Vivo

**Figure 9:**
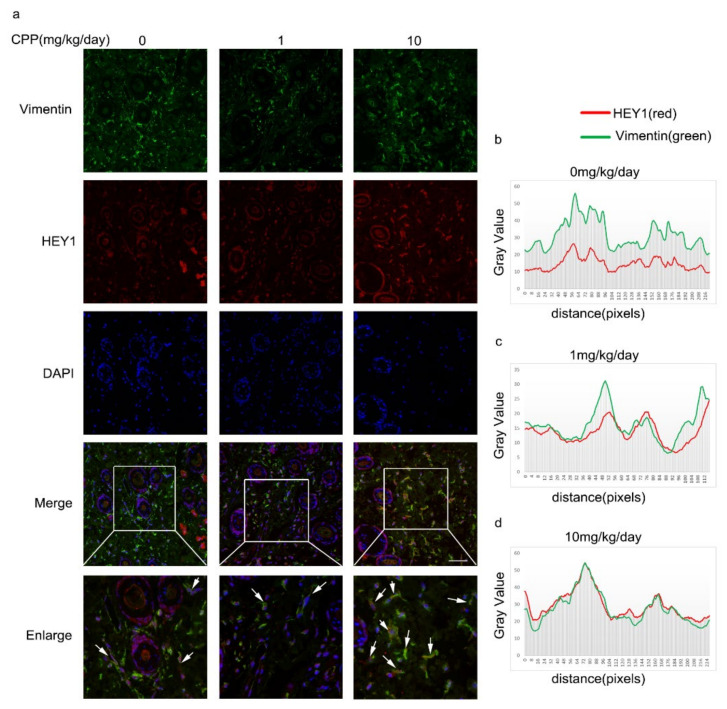
**CPP increased the protein levels of HEY1 in HDFs in vivo.** (**a**) After two weeks of continuous intraperitoneal injection of mice with different concentrations of 1 mg/kg/day and 10 mg/kg/day, of CPP and PBS, respectively, mouse dorsal skin was extracted, paraffin sections were performed, and the protein levels of HEY1 and Vimentin were detected by immunofluorescence. White arrows indicate cells expressing both Vimentin and HEY1. Scale bars: 100μm. (**b**–**d**) ImageJ analysis of co-localization of Vimentin and HEY1.

To further prove that CPP induces HDFs to differentiate into VECs by promoting the expression of HEY1 in HDFs, we injected different concentrations of CPP (1 mg/kg/day, 10 mg/kg/day) into mice intraperitoneally. After two weeks of treatment, we found that CPP wasn’t significantly toxic *in vivo* (Fig.S2). Next, we extracted mouse dorsal skin, and performed paraffin sections and immunofluorescence. We labeled HDFs with Vimentin and detected the protein level of HEY1 in Vimentin-positive HDFs. We found that the level of HEY1 was significantly increased in the CPP-injected group compared with the PBS-injected group (Figure 9a), and the co-localization of HEY1 with Vimentin was increased (Figure 9b–d). These data demonstrated that CPP also promoted the accumulation of HEY1 in HDFs in vivo.

**Figure 10:**
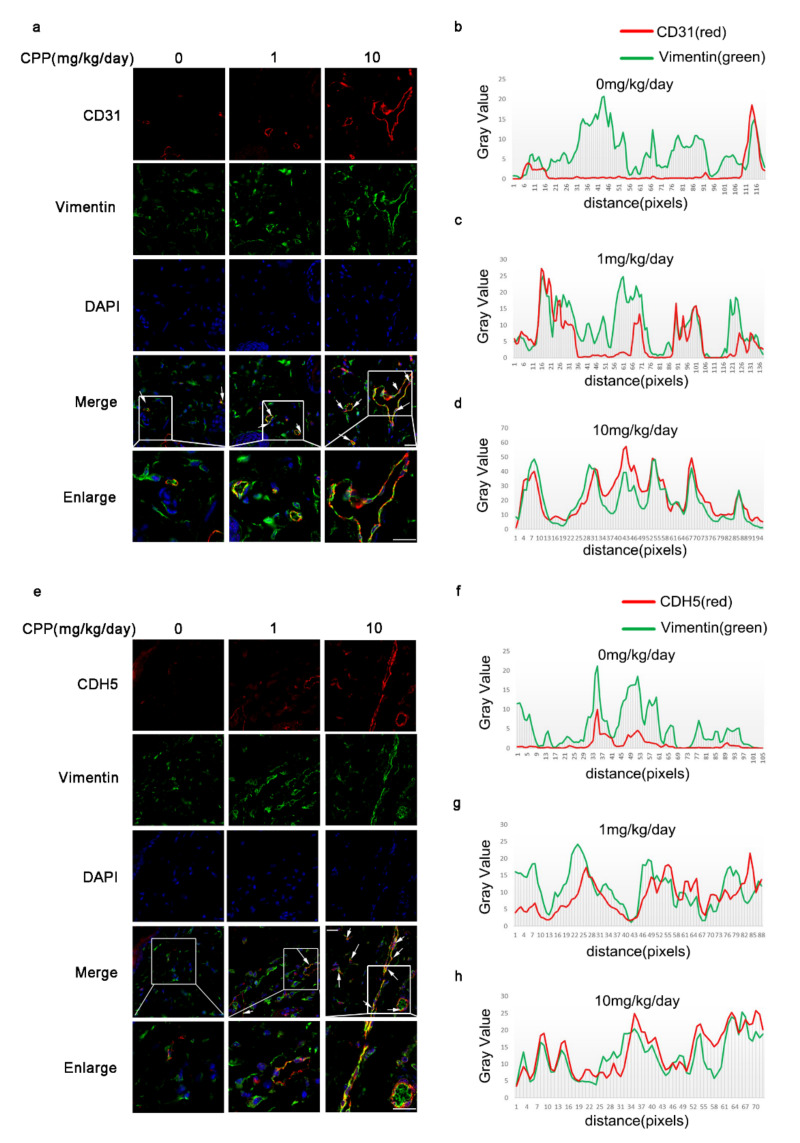
**CPP increased the protein levels of CD31 and CDH5 in HDFs in vivo.** (**a**) and (**e**): After two weeks of continuous intraperitoneal injection of mice with different concentrations (1 mg/kg/day, 10 mg/kg/day) of CPP and PBS, paraffin sections from mouse dorsal skin were performed, and immunofluorescence was conducted to detect the co-localization of CD31 and Vimentin or CDH5 and Vimentin. White arrows indicate cells expressing both Vimentin and HEY1. Scale bars: 100μm. (**b**–**d**) and (**f**–**h**): ImageJ analysis the co-localization of different proteins.

To further demonstrate whether CPP can promote the protein expression of CD31 and CDH5 in HDFs while increasing HEY1 levels in vivo, we conducted immunofluorescence to detect the co-localization of Vimentin and CD31, and Vimentin and CDH5. Consistently, the results showed that the co-localization of Vimentin and CD31, and Vimentin and CDH5, were up-regulated (Figure 10). Taken together, these data indicated that CPP could up-regulate the protein levels that maintain endothelial cell function in HDFs in vivo.

## 4. Discussion

Small chemical molecules can regulate cell phenotypes by targeting signaling pathways, epigenetic modifications, and metabolic processes [23,24]. Previous studies proved that CPP could induce HDFs to differentiate into vascular endothelial cells with a high differentiation rate. Researchers are increasingly using different methods to induce HDFs to differentiate into VECs [9,25,26], but the differentiation mechanism has been unclear. Here, we reported that CPP could bind to HOCl and inhibit the oxidative modification of proteins, thereby inducing HDFs to differentiate into vascular endothelial-like cells. Simultaneously, CPP, as a fluorescent probe, has the advantage of auto-fluorescence in cells and becomes a powerful tool for studying the detailed molecular mechanism of cell differentiation. Using the fluorescent characteristics of CPP, we could directly assess the co-localization of CPP and related proteins through immunofluorescence.

HOCl generated during the oxidation of Cl^−^ by H_2_O_2_ produced by myeloperoxidase (MPO) mainly occurs inside activated neutrophils [27]. Studies have shown that endogenous HOCl can be used as an essential signaling molecule to regulate many critical physiological processes, such as apoptosis, atherosclerosis, chronic inflammation, etc., [28,29,30]. However, it is unclear whether HOCl plays a role in the differentiation of dermal fibroblasts into vascular endothelial-like cells. CPP can bind HOCl to inhibit the effects of HOCl, and has become a valuable tool for studying the impact of HOCl on the differentiation of HDFs into vascular endothelial cells. In this study, experiments using the co-localization of CPP and PHD2 showed that CPP targeted PHD2. We also found that CPP inhibited the enzyme activity of PHD2 during the CPP-induced differentiation of HDFs into vascular endothelial-like cells. In contrast, HOCl activated PHD2. When HDFs were treated with CPP and HOCl, CPP no longer inhibited the enzyme activity of PHD2. PHD2 is considered to be the main down-regulating factor of HIF-1α during normoxia, therefore, it plays an important role in regulating cell differentiation. Studies have shown that conditional deletion of the PHD2 gene in chondrocytes promoted the differentiation of progenitors into hypertrophic chondrocytes [31]. In previous studies, the HOCl-induced oxidation of proteins led to changes in protein functions and cell fates [32,33,34]. We mutated the three oxidatively modified cysteines in the dioxygenase domain of PHD2, and found that the mutation at Cys302 disrupted the ability of CPP to induce HDFs to differentiate into vascular endothelial cells [13,35]. Simultaneously, we found that the mutation at Cys302 caused CPP to fail to inhibit the enzyme activity of PHD2 and to promote the increased level of HIF-1α protein. Therefore, our study is the first to demonstrate that CPP inhibits the oxidative modification of PHD2 at Cys302 by binding HOCl. Other studies have reported that the proline mutation to arginine at position 317 of PHD2 significantly reduces its enzymatic activity. In addition, residues A371, H374 and R383 are also vital to maintaining PHD2 function [21,36]. Here, we report that residue Cys302 of PHD2 is also essential to inhibiting enzyme activity.

Studies have shown that prolyl hydroxylase domain-containing enzyme (PHD) isoform 2 (EGLN1) can hydroxylate HIF-1α, thereby promoting the degradation of HIF-1α [37]. Hypoxia-inducible factor (HIF-1) is a heterodimeric transcription factor protein consisting of HIF-1α and HIF-1β domains. However, under hypoxic conditions, HIF-1α can remain stable and, as a transcription factor, enters the nucleus to regulate the expression of related angiogenic genes, thereby promoting angiogenesis [10,38]. However, HIF-1α regulates cell fate differently in different cells. Studies have shown that HIF-1 involves multiple aspects of tumor progression, such as metastasis, angiogenesis, and immune evasion [39]. However, in recent years, more and more studies have found that hypoxia is critical for stem cell differentiation into endothelial cells, which is inseparable from the fact that hypoxia promotes the binding of HIF-1α to its angiogenesis-related target genes [19,40,41]. Thus, the role of HIF1a in regulating cell fate is uncertain. Here, we found that CPP can significantly promote the increase in HIF-1α protein level and the expression of pro-angiogenic factors, which further indicates that HIF-1α can regulate the differentiation of HDFs into VECs. In addition, we also tested the mRNA level of HIF-1α and found that its mRNA level was also elevated. Interestingly, experiments using cycloheximide, a protein synthesis inhibitor, showed that CPP still increased the protein level of HIF-1α, which suggests that CPP promotes the increase in HIF-1α levels by inhibiting the enzyme activity of PHD2. However, in this experiment, we observed that after HDFs were treated with CHX, although CPP could still increase the protein level of HIF-1α, it did not recover to the original level. We think the reason was that HIF-1 promoted the expression of its α-subunit in an epigenetically regulated transactivation loop [42]. Therefore, we inferred that CPP promoted the increase in the mRNA level of HIF-1α due to the regulation of HIF-1.

Studies have shown that HIF-1α can induce the expression of HEY1 [43,44,45,46]. HEY1 is a crucial transcription factor for the maintenance of VECs [47]. Researchers have used HEY1 and other transcription factors to directly reprogram HDFs into VECs [25]. Consistently, studies have found that the knockout of HEY1 in the VECs of mice can cause abnormalities in the structure of large blood vessels, while HEY1 double knockout with HEY2 can cause severe mortality [15,48]. Similarly, researchers found that the expression of HEY1 is elevated during the differentiation of endothelial progenitor cells into arterial endothelial cells [49]. However, although studies have shown a loss of expression of the arterial endothelial markers CD44, neuropilin1 and ephrinB2 in Hey1/Hey2 KO mice, little research has been conducted on HEY1 targets. In the study of HEY1 promoting angiogenesis, researchers found that HEY1 can inhibit the proliferation and migration of endothelial cells by inhibiting the expression of VEGF, thereby involving induction and/or maintenance of the mature, quiescent vascular phenotypes [47]. In our research, we found that CPP could induce the expression of HEY1, and knockdown of HEY1 prevented CPP from inducing the differentiation of HDFs to form VECs, which further demonstrated that HEY1 played a crucial role in the differentiation of HDFs into vascular endothelial cells. To prove that CPP can also play a role in vivo, we used Vimentin (+) to label HDFs and found that CPP could also promote the expression of HEY1 in HDFs in vivo. In addition, we observed that HEY1 contained other equally strong bands around 40 kDa and 55 kDa in Western blot, and we suspected post-translational modification, such as sumoylation, since sumoylation of nucleoproteins is most common [50].

## 5. Conclusions

**Figure 11:**
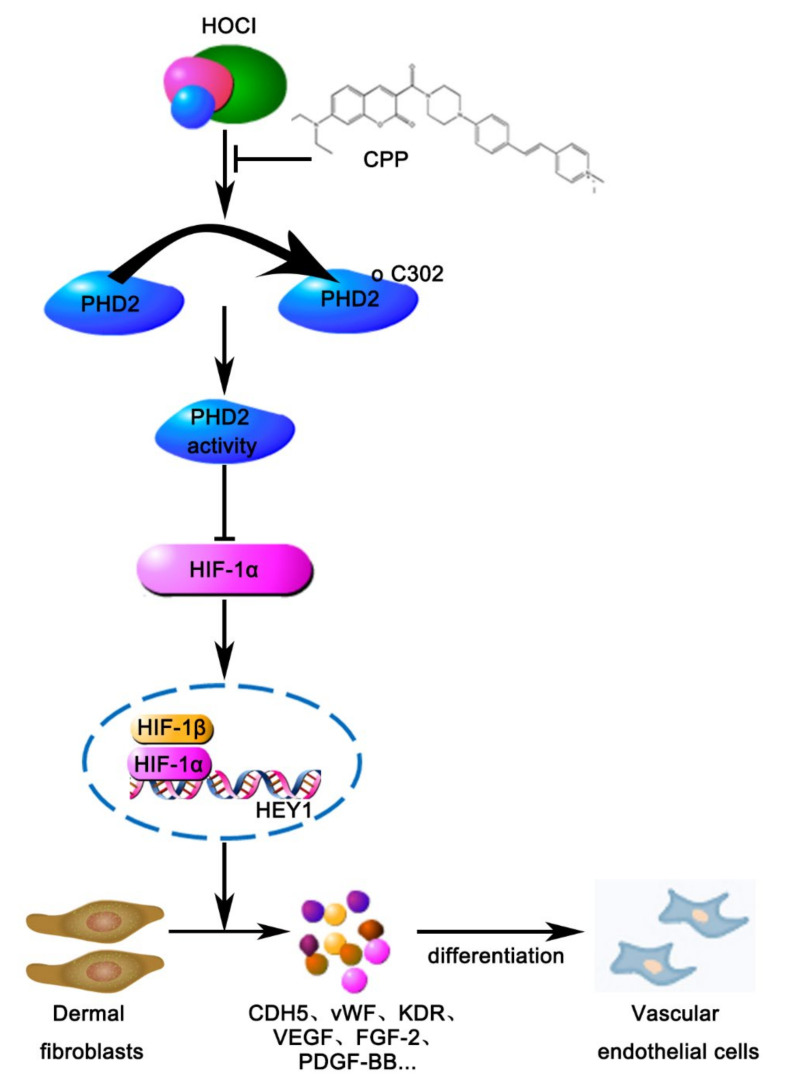
**The mechanism of CPP-induced differentiation of HDFs into VECs.** CPP can bind to HOCl, inhibiting the oxidation of PHD2 at cysteine 302, thereby inhibiting the activity of PHD2. The inhibition of PHD2 enzyme activity promotes the increased protein level of HIF-1α, which promotes the expression of HEY1, therefore, increases the expression of VEGF, FGF-2 and PDGF-BB, and induces the differentiation of HDFs into VECs.

In summary, we discovered that the HOCl probe CPP reduces HOCl levels to inhibit PHD2 activity through regulation of Cys302, which results in an increased HIF-1a level that efficiently induces the expression of HEY1 and the differentiation of HDFs into VECs (shown schematically in Figure 11). Therefore, these results provide new ideas for the study of HDFs differentiation, and also provide a new mechanism of hypochlorous acid in the differentiation process of HDFs into VECs.

## Data Availability

All data generated or analyzed during this study are included in this published article. RNA-seq generated datasets are available in the NCBI repository BioProject accession number of RNA-seq is PRJNA849353.

## Supplementary Materials

The following supporting information can be downloaded at https://www.mdpi.com/article/10.3390/cells11193126/s1. Figure S1: Verification of the interference efficiency of two small interfering RNAs on HEY1 in HDFs cells. Figure S2: In vivo toxicology experiment. Table S1: The qPCR primers.
