# Supplementary material for: HOCI Probe CPP Induces the Differentiation of Human Dermal Fibroblasts into Vascular Endothelial Cells through PHD2/HIF-1α/HEY1 Signaling Pathway
Source: Cells. 2022 Oct 4;11(19):3126. doi: 10.3390/cells11193126 (PMC9562224; doi:10.3390/cells11193126)
Supplement: Supplementary file 1 [file cells-11-03126-s001.zip › cells-1897727-supplementary.pdf]

SUPPLEMENTARY FIGURES and FIGURE LEGENDS

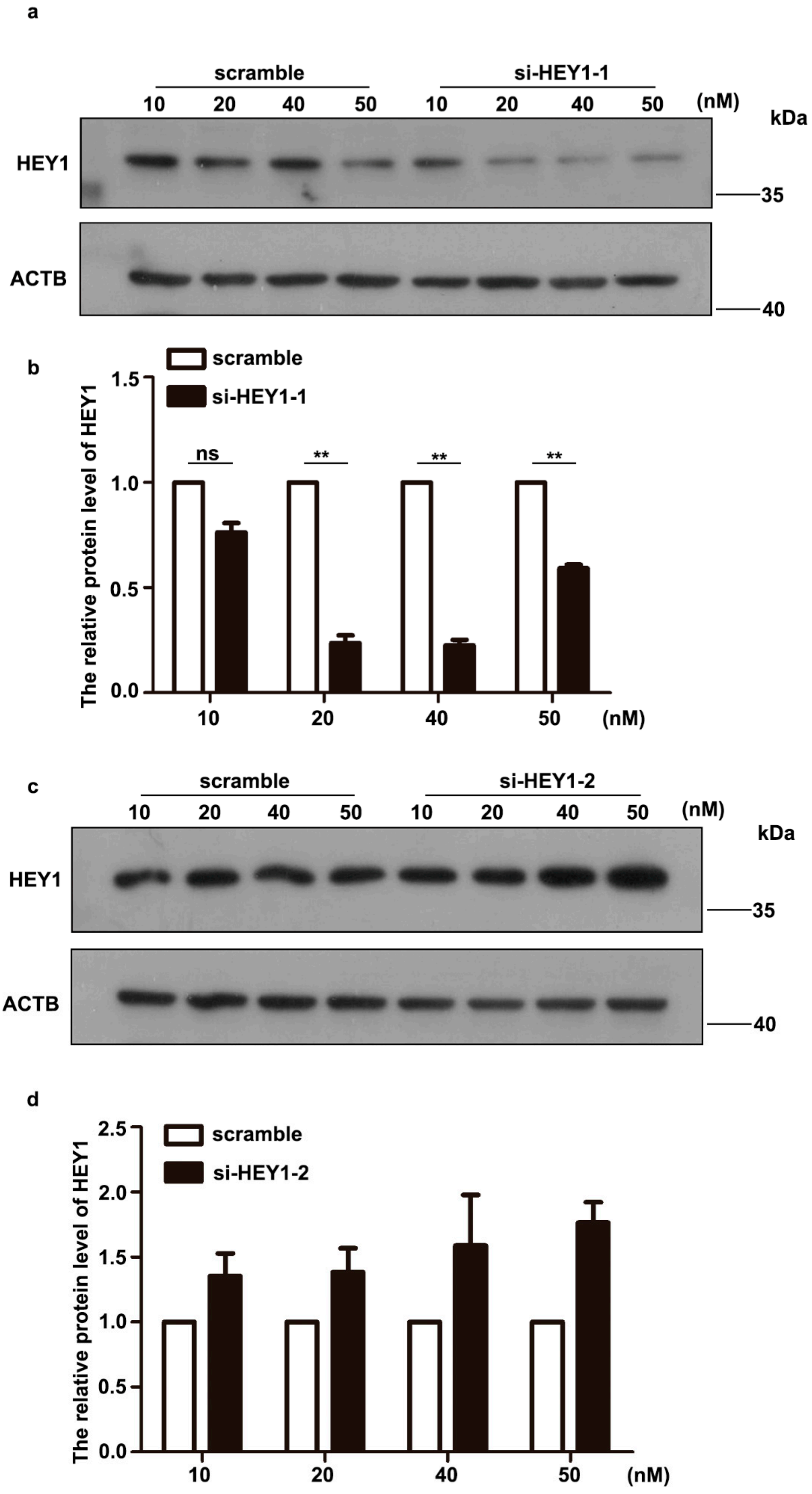

Figure S1. (a,b) and (c,d) Verification of the interference efficiency of two small interfering RNAs on HEY1 in HDFs cells. Data are presented as means ± SEM, *p < 0.05, **p < 0.01, ***p < 0.001, n = 3.

a

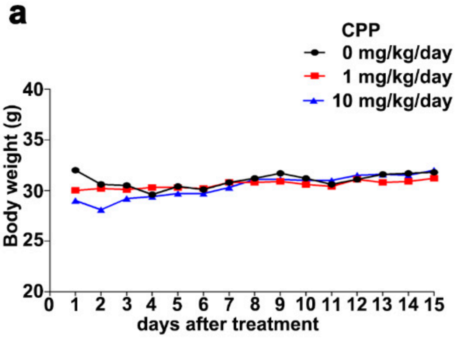

b

Table 1. Effect of CPP with various dosages on organ coefficients of mice(x̄±s, n=3)

CPP dose (mg/kg)	Heart	Liver	Spleen	Lung	kidney	Brain	Pancreas	Thymus
0	0.57±0.10	5.11±0.41	0.34±0.09	0.62±0.01	1.36±0.03	1.53±0.09	0.99±0.28	0.27±0.05
1	0.67±0.22	5.80±0.92	0.40±0.17	0.72±0.04	1.82±0.27	1.43±0.09	1.16±0.06	0.39±0.07
10	0.49±0.02	5.16±0.09	0.40±0.16	0.66±0.07	1.50±0.02	1.39±0.02	1.24±0.42	0.23±0.08

c

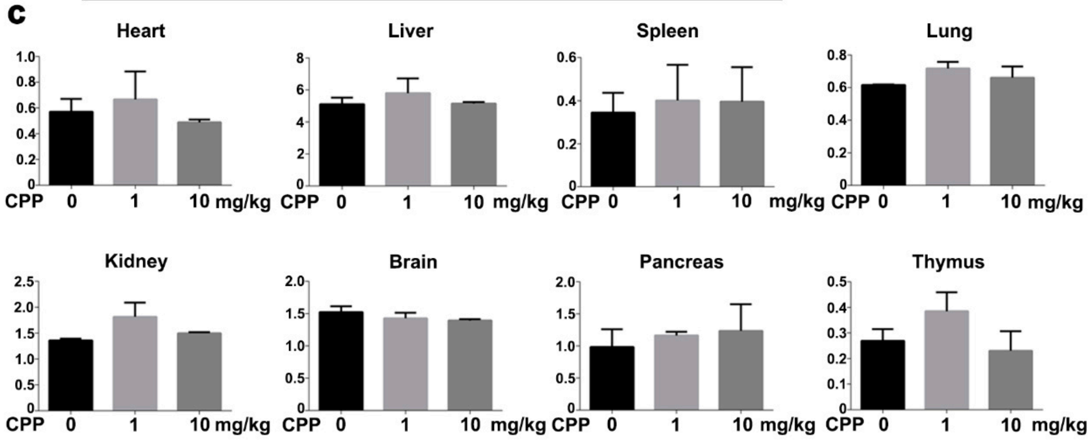

Figure S2. In vivo toxicology experiment. Mice were injected intraperitoneally with CPP (0 mg/kg/day, 1 mg/kg/day or 10 mg/kg/day) for two weeks (D14), and then the body weights (a) and organ coefficients (b,c) were measured.

Table S1. the qPCR primers.

Primers	FORWARD	REVERSE
CD31	5′-TCAGACGTGCAGTACACGGA-3′	5′-CTTTCCACGGCATCAGGGAC-3′
CD133	5′-GTGGCGTGTGCGGCTATGAC-3′	5′-CCAACTCCAACCATGAGGAAGACG-3′
Vimentin	5′-GGTGGACCAGCTAACCAACG-3′	5′-TTGCAGGGTGTTTTCGGCTT-3′
VEGF	5′-ATCGAGTACATCTTCAAGCCAT-3′	5′-GTGAGGTTTGATCCGCATAATC-3′
FGF-2	5′- CATCAAGCTACAACTTCAAGCA-3′	5′-CCGTAACACATTTAGAAGCCAG-3′
PDGF-BB	5′-ACCGCACCAACGCCAACTTC-3′	5′-TCTTCCGCACAATCTCGATCTTTCTC-3′
CDH5	5′- AAAGAATCCATTGTGCAAGTCC-3′	5′- CGTGTTATCGTGATTATCCGTG-3′
eNOS	5′-GATGTTACCATGGCAACCAAC-3′	5′-GAAAATGTCTTCGTGGTAGCG-3′
ANGPT1	5′- GGGAGGTTGGACTGTAATACAA-3′	5′-TGTCATACTGTGAATAGGCTCG-3′
vWF	5′-CCTGTTACTATGACGGTGAGAT-3′	5′-CATGAAGCCATCCTCACAGTAG-3′
Collagen1	5′- AAAGATGGACTCAACGGTCTC-3′	5′- CATCGTGAGCCTTCTCTTGAG-3′
FAP	5′-GAGTCCAGAATGTTTCGGTCCTGTC-3′	5′- AACAAAGAATCCACCAGCCCATCC-3′
FSP1	5′- GACTGGATTCATTGGTTTGGAC-3′	5′- CTCCAGAGTACCGGATATATGC-3′
ACTA2	5′-CTTCGTTACTACTGCTGAGCGTGAG-3′	5′- CCCATCAGGCAACTCGTAACTCTTC-3′
HIF1-α	5′-AAGTGTACCCTAACTAGCCG-3′	5′-TCACAAATCAGCACCAAGC-3′
HEY1	5′-TGGCAGAAGTTGCGCGTTAT-3′	5′-CGCTGGGAAGCGTAGTTGTT-3′
β-Actin	5′- CCTGGCACCCAGCACAAT-3′	5′-GCCGATCCACACGGAGTACT-3′
